# Provision of care through telemedicine during a natural disaster: a case study

**DOI:** 10.1038/s41394-020-0309-2

**Published:** 2020-07-09

**Authors:** Elizabeth C. Pasipanodya, Kazuko Shem

**Affiliations:** 1 Rehabilitation Research Center, Santa Clara Valley Medical Center, San Jose, CA USA; 2 Department of Physical Medicine and Rehabilitation, Santa Clara Valley Medical Center, San Jose, CA USA

**Keywords:** Rehabilitation, Health policy

## Abstract

**Introduction:**

As the frequency and intensity of natural disasters increases, disaster preparedness is necessary for individuals with disabilities, including those with spinal cord injury (SCI). However, despite vulnerability to poorer outcomes, disaster preparedness for individuals with SCI is inadequate. Telemedicine has been effectively used to mitigate the impact of natural disasters. In this case study, we describe the provision of clinical care, via telemedicine, to an individual with SCI who was affected by the California Valley Fire in 2015.

**Case presentation:**

The individual described was an adult who sustained a SCI. Before discharge from acute inpatient rehabilitation, they enrolled in a research study, through which they received outpatient SCI-specific care via telemedicine (teleSCI). The participant attended several teleSCI visits prior to the start of the Valley Fire. In the midst and immediate aftermath of prolonged wildfire, and despite experiencing significant personal loss as a result of the fire, the participant continued to receive teleSCI services. TeleSCI was used to address emergent and extant medical concerns and to facilitate replacement of medical supplies and equipment destroyed by fire.

**Discussion:**

TeleSCI was used to provide continuity of care and was responsive to the needs of an individual with SCI severely affected by wildfire. Emergency preparedness that meets the needs of persons with disabilities is critical during times of crisis. Telemedicine may be an effective tool that can be applied to meet the medical needs of individuals with SCI and to mitigate the impact of disasters.

## Introduction

Disaster preparedness is increasingly critical as the severity and impact of natural disasters grows as a result of global climate change [1–7]. Coinciding with the rise in average temperatures, more earthquakes, tornadoes, hurricanes, and wildfires causing significant property damage and loss of life have been recorded in the United States in recent decades than previous ones [1, 3, 8–10]. Although disaster preparedness to mitigate the negative impact of natural disasters is vital for every person, it is particularly so for individuals with disabilities, including those with spinal cord injury (SCI), who are more vulnerable during crises [11–13].

During natural disasters and other crises, persons with SCI encounter greater barriers to mobility and to maintaining their physical and mental health. For instance, the majority of US individuals with SCI rely on wheelchairs or scooters; however, natural disasters typically result in greater mobility challenges such that individuals with SCI may experience difficulties with evacuation, transportation, and access to customized living quarters [14, 15]. Furthermore, disasters may result in disruptions to receiving healthcare. Given the high degree of psychiatric comorbidities (e.g., depression, anxiety, and post-traumatic stress disorder) among individuals with SCI and their unique physical health concerns (e.g., spasticity and chronic pain), the stress and disruption caused by natural disasters may exacerbate physical and mental health conditions among individuals with SCI [16–21]. However, despite vulnerability to poorer outcomes in the context of natural disasters, studies examining disaster preparedness among individuals with SCI and other disabilities suggest inadequate planning and a lack of specific strategies for evacuation, survival, and care needs [22–25].

Telemedicine is the provision of clinical services remotely, through telecommunications-based networks, usually to individuals who might otherwise be restricted from accessing healthcare due to environmental constraints [26]. The use of telemedicine for routine encounters has steadily increased and a review of telemedicine research studies suggests efficacy for treatment and follow-up among individuals with SCI [27]. As telemedicine enables healthcare visits when in-person visits would be otherwise inconvenient or impossible, its relevance for disaster preparedness and mitigation is clear. Indeed, telemedicine has been used to provide care and to support relief efforts during disaster situations [28, 29]. In this case study, we describe the provision of clinical care, via telemedicine, to an individual with SCI who was affected by the Valley Fire in 2015. To date, the Valley Fire is recorded as the 5th most destructive fire in the state of California, burning 76,067 acres of land, destroying 1955 buildings, and causing 4 deaths from its start on September 12, 2015 till its containment on October 15, 2015 [30].

## Case presentation

The individual described in this case report was an adult who sustained a complete traumatic SCI several months before the 2015 Valley Fire. They underwent acute inpatient rehabilitation at Santa Clara Valley Medical Center (SCVMC) and, at discharge, were independent in most activities of daily living including wheelchair transfers and maneuverability. The individual with SCI also followed an intermittent catheterization program for bladder management and a bowel management program consisting of laxatives and stool softeners with digital stimulation and evacuation. Prior to returning to their private residence in the north central portion of California, a significant distance away from SCVMC, they enrolled in a telemedicine for SCI (teleSCI) study (SCiPad; Craig H. Neilsen Foundation Quality of Life Grant #296161), through which they received remote SCI-specific care from a board-certified SCI specialist. As part of the study, the participant received a tablet (Apple iPad Air) with a 6-month cellular data plan as well as a home blood pressure machine for self-monitoring [31, 32]. The participant’s teleSCI visits were completed through the FaceTime application. FaceTime uses end-to-end encryption to prevent interception and decryption of data while in transit between devices, satisfying the Health Insurance Portability and Accountability Act standards for the protection of electronic health information [33]. TeleSCI visits with the physician were able to be scheduled within a day of request and a program coordinator, who was available to all participants for nonemergency needs during regular business hours, liaised between the participant and the physician outside of scheduled appointments. All applicable institutional and governmental regulations concerning the ethical use of human volunteers were followed during the SCiPad study.

Prior to the Valley Fire, the participant attended several teleSCI visits with the physician; visits typically addressed comorbidities and concerns common among individuals with SCI (e.g., neurogenic bladder, pressure sores, urinary tract infections, and deep vein thrombosis) [20, 21]. Using FaceTime’s video capabilities, the physician could see and hear the participant, allowing visual and auditory aids that were helpful in informing the physician’s assessment of the participant’s physical and mental well-being. Blood pressure readings were also taken by the participant and reported to the physician at each teleSCI visit. In addition, as the participant received primary care near their place of residence and had routine lab work, test results and participant notes were reciprocally available to the SCI physician and the participant’s primary care doctor for coordination of care.

Within days of the start of the Valley Fire, the participant attended a scheduled teleSCI visit and they reported losing their home to wildfire and temporarily moving in with their significant other, whose residence remained intact. The participant additionally reported having to live with other acquaintances and family members who were similarly displaced by the fire. Furthermore, the participant reported that although they still had their manual wheelchair, most of their supplies and durable medical equipment (DME), including commode/shower bench, electric compression devices, standing frame, and equipment for home exercise were lost. Thus, the participant’s new medical needs at that time were to replace essential DME and supplies; these were immediately ordered and promptly authorized by the participant’s insurance. In addition to addressing pressing concerns, existing concerns were reviewed and follow-up teleSCI appointments were scheduled.

In between teleSCI visits, and while efforts to contain the Valley Fire continued, the participant communicated with the research coordinator and physician via secure email regarding their physical health concerns, including lower extremity swelling and pressure sores, and the participant provided images for the physician to review. A prescription to replace heel float boots and stretching straps lost in the fire was provided to the participant and they were instructed to wear heel protectors and to continue to monitor areas with active pressure sores. The participant additionally corresponded with the physician regarding obtaining authorization for a vehicle modified for a driver with disabilities. Subsequent to this, follow-up teleSCI visits discussed routine participant concerns and monitored participant mood during this period of high stress. Within six months of the start of the fire, the participant relocated to a new house and re-established their lifestyle.

## Discussion

This case report describes the use of teleSCI to provide care to an individual with SCI in the midst and immediate aftermath of a natural disaster. As a result of the Valley Fire, the individual with SCI lost their home, most of their possessions, and the majority of their adaptive devices and medical supplies. However, despite their experience of upheaval to their daily life and significant personal loss, they were able to seamlessly receive care that was responsive to extant and emergent needs through telemedicine services.

As a result of climate change, natural disasters are occurring more frequently with more negative effects to human lives and settlements [3–7, 34, 35]. Individuals with disabilities are at higher risk of injury and death following natural disasters as consequence of factors that include impaired mobility and higher medical comorbidities [36, 37]. As illustrated by this case example, when individuals with SCI experience a natural disaster, they may also face the loss of DMEs, supplies, and medications they routinely use, resulting in poorer health outcomes. Furthermore, their caregivers and their primary care providers, who live and practice in the same area, may also be affected by the disaster; thus, individuals with SCI may be unable to access and receive adequate or expeditious care. Given additional hurdles experienced by individuals with SCI, teleSCI, as an adjunct to other disaster relief services, may provide a means of delivering timely and convenient care and may facilitate swifter replacement of necessary equipment and supplies.

Research suggests that telemedicine can be an efficacious and cost-effective modality for providing care and that it may also have measurable benefits to patients, reducing the time and financial costs of attending in-person visits [38–43]. In disaster situations, a salient advantage of telemedicine is the ability to utilize remotely located providers when local and in-state resources are scarce or overwhelmed [44]. Indeed, telemedicine may circumvent sole reliance on health systems in affected areas and, for individuals with SCI and other disabilities, it may also limit travel, through potentially unsafe conditions, to seek medical care.

Recommendations have been made to have telemedicine “vigorously applied to solve medical needs in extreme and disruptive environments” [28]. In particular, recommendations suggest establishing infrastructure for telemedicine programs, principally in areas that are at high risk for disasters, so that when emergency situations are encountered, existing telemedicine networks can be used [45]. Indeed, reviews of several natural disasters suggest that instituting telemedicine capabilities after a disaster, rather than using extant infrastructure in response to disasters, is costly and may delay the provision of care [28, 29, 45]. A good example of the benefits of co-opting existing telemedicine programs during disasters is provided by the University of Texas Medical Branch’s response to Hurricane Ike in 2008. Although Hurricane Ike resulted in significant disruptions to the medical center’s operations, telemedicine services resumed significantly earlier and at higher levels than in-person care in the immediate aftermath of the hurricane [46]. Moreover, in another example, following a 7.6 magnitude earthquake in Pakistan in 2005, mobilization of resources at Rawalpindi’s telemedicine training programs ameliorated effects of the disaster by enabling communications between affected rural clinics and preventing clinics from becoming overwhelmed [28, 47]. In our case example, an existing telemedicine service was used to provide timely and responsive care to an individual impacted by the Valley Fire within days of the start of the wildfire and before it was contained.

Apart from response to natural disasters, telemedicine has a critical role in emergency response more broadly. Indeed, the need for telemedicine and its wider integration into healthcare systems has become even more salient following the coronavirus 2019 (COVID-19) pandemic [48]. As a result of COVID-19’s high transmissibility, recommendations were made to limit in-person clinic visits and to find alternatives to face–face encounters, including using telemedicine for visits [49]. However, changes to reimbursement structures had to be made to accommodate the rapid shift to telemedicine services. In particular, the Centers for Medicare and Medicaid Services temporarily expanded Medicare reimbursement to allow healthcare seekers to receive telemedicine services regardless of their location. Prior to this, Medicare reimbursed telemedicine only in limited circumstances (e.g., when healthcare seekers resided in a designated rural area and when they received telemedicine services at a medical facility) [50]. Although the use of telemedicine has grown rapidly in recent years, a combination of a number of barriers, including limited financial reimbursement, have resulted in its relatively slow uptake nationally [51–53].

In summary, scaling-up telemedicine service and incorporating routine telemedicine systems into disaster preparedness and mitigation plans may increase the likelihood of successful response to disasters. Given current research that suggests that emergency preparedness is relatively low among persons with SCI and other disabilities, a multipronged approach that incorporates disaster preparedness education and investment in infrastructure (including telemedical) that can be adaptively used to meet needs during natural disaster may improve post-disaster outcomes [22, 23, 25].
